# Chest ultrasound for evaluation of bilateral pulmonary infiltrates in initensive care unit: A comparison with clinical assessment, sonographic assessment

**DOI:** 10.1186/2197-425X-3-S1-A613

**Published:** 2015-10-01

**Authors:** J Jeon, SB Hong

**Affiliations:** Asan Medical Center, University of Ulsan College of Medicine, Seoul, Korea Republic of

## Background

When the new bilateral air space pacification develops, we are usually indistinguishable bilateral pulmonary infiltrates from congestive cardiac failure. Until now, for the differential diagnosis of cardiogenic edema and pneumonia, laboratory finding including white blood cell (WBC), C-reactive protein (CRP), B-type natriuretic peptide (BNP) and imaging studies such as chest computed tomography (CT) and/or echocardiography were have been used. We evaluated whether chest ultrasound (CUS, heart+lung) could assist in the differential diagnosis of pulmonary edema and pneumonia. So, we conducted a comparison between clinical diagnosis (C-Dx) with the result of the laboratory finding and sonographic diagnosis (US-Dx) with CUS.

## Method

We performed a retrospective observational study evaluating the utility of CUS in helping to differentiate pulmonary edema and pneumonia who were admitted to the ICU from January 2013 to December 2014. The patients who admitted to the medical intensive care unit (MICU), of the patients showed new developed bilateral infiltration on chest X-ray, targeting patients who underwent CUS. CUS was performed in all patients on the first day of admission and after having obtained a clinical diagnosis of disease. We compared the US-Dx is determined based on the cardiac and lung ultrasound with C-Dx is determined on the basis of WBC, CRP, BNP. WBC > 11,000/uL, CRP > 1 mg/dL and BNP > 300 pg/mL were interpreted as a meaningful. C-Dx and US-Dx were compared to see if it matches divided into three categories of pneumonia, pulmonary edema, combined.

## Result

During the 2-years study period, seventy-seven patients were enrolled. C-Dx was associated with US-Dx (P < 0.00, Fisher's exact test). US-Dx was associated with final diagnosis (P < 0.00, Fisher's exact test).

## Conclusion

In critically ill patients, diagnosis using chest ultrasound and diagnosis was inferred to be the result of laboratory test substantially coincided. Therefore, using such a point, it is useful for the differential diagnosis of the patient.

